# Differentiation of Parkinson’s disease and Parkinsonism predominant multiple system atrophy in early stage by morphometrics in susceptibility weighted imaging

**DOI:** 10.3389/fnhum.2022.806122

**Published:** 2022-08-02

**Authors:** Qingguo Ren, Yihua Wang, Xiaona Xia, Jianyuan Zhang, Cuiping Zhao, Xiangshui Meng

**Affiliations:** ^1^Department of Radiology, Qilu Hospital (Qingdao), Cheeloo College of Medicine, Shandong University, Qingdao, China; ^2^Department of Neurosurgery, Qilu Hospital (Qingdao), Cheeloo College of Medicine, Shandong University, Qingdao, China; ^3^Department of Neurology, Qilu Hospital (Qingdao), Cheeloo College of Medicine, Shandong University, Qingdao, China

**Keywords:** multiple system atrophy, Parkinson’s disease, susceptibility weighted imaging, early duration, diametral measurement

## Abstract

**Background and purpose:**

We previously established a radiological protocol to discriminate multiple system atrophy-parkinsonian subtype (MSA-P) from Parkinson’s disease (PD). However, we do not know if it can differentiate early stage disease. This study aimed to investigate whether the morphological and intensity changes in susceptibility weighted imaging (SWI) of the lentiform nucleus (LN) could discriminate MSA-P from PD at early stages.

**Methods:**

We retrospectively enrolled patients with MSA-P, PD and sex- and age-matched controls whose brain MRI included SWI, between January 2015 and July 2020 at the Movement Disorder Center. Two specialists at the center reviewed the medical records and made the final diagnosis, and two experienced neuroradiologists performed MRI analysis, based on a defined and revised protocol for conducting morphological measurements of the LN and signal intensity.

**Results:**

Nineteen patients with MSA-P and 19 patients with PD, with less than 2 years of disease duration, and 19 control individuals were enrolled in this study. We found that patients with MSA- P presented significantly decreased size in the short line (SL) and corrected short line (cSL), ratio of the SL to the long line (SLLr) and corrected SLLr (cSLLr) of the LN, increased standard deviation of signal intensity (SIsd_LN, cSIsd_LN) compared to patients with PD and controls (*P* < 0.05). With receiver operating characteristic (ROC) analysis, this finding had a sensitivity of 89.5% and a specificity of 73.7% to distinguish MSA- P from PD.

**Conclusion:**

Compared to PD and controls, patients with MSA-P are characterized by a narrowing morphology of the posterior region of the LN. Quantitative morphological changes provide a reference for clinical auxiliary diagnosis.

## Introduction

Multiple system atrophy (MSA) is one of the atypical parkinsonian disorders and is a sporadic, middle-age onset disorder with two subtypes, the MSA-parkinsonian subtype (MSA-P) and the MSA-cerebellar subtype (MSA-C), because of selective atrophy and neuronal loss in the striatonigral and olivopontocerebellar systems (Fanciulli et al., 2019; Marsili et al., 2019). MSA-P is a fatal neurodegenerative disease with autonomic failure and Parkinsonian and/or cerebellar features that are classified as Parkinsonian and cerebellar subtypes. MSA-P and Parkinson’s disease (PD) have similar clinical presentations, such as rigidity, bradykinesia, and response to levodopa, making it challenging in clinical practice for differential diagnose at an early stage (2–3 years of disease duration). It is very important to discriminate MSA-P from PD in the early stage to accurately predict prognosis in terms of disability, response to therapy, and survival. Conventional magnetic resonance imaging (MRI) has been used extensively to distinguish between MSA-P and PD. The hyperintense rim of the lateral putaminal margin and hypointensity of the posterolateral putamen on routine (fluid-attenuated inversion recovery) FLAIR and/or T2 imaging have assisted physicians in the differential diagnosis of MSA-P and PD (Schrag et al., 2000). However, these are subjective, not found in all patients with MSA-P, and have low sensitivity, especially in the early stage (Meijer et al., 2012). Hypointense putaminal signal changes on T2*- or susceptibility-weighted imaging are relatively specific for MSA-P (Yoon et al., 2015; Heim et al., 2017). However, these changes are subjective. Recently, several reports have semi- quantitatively investigated signal intensity changes in the lentiform nucleus (LN) (Seppi et al., 2003; Meijer et al., 2015a,b). In a study from Hwang (Hwang et al., 2015), quantitative measurement of the putaminal width was performed, but the selection of the representative section was subjective. In our previous work, we defined standard axis plane, vertical line to define the anterior-posterior location of the LN and established a radiological protocol to perform morphological measurements of the LN and signal intensity, which could discriminate MSA-P from PD with a sensitivity of 94.7% and specificity of 63.2% (Ren et al., 2020). However, we do not know the applicability of this method for disease differentiation in the early stage of MSA-P. The present study aimed to investigate this morphological and intensity measurements for discriminating MSA-P from PD and healthy individuals within 2 years of disease duration.

## Materials and methods

### Ethical approval and subject description

This study was approved by the ethics committee of Qilu Hospital and was performed in accordance with the Helsinki Declaration of the World Medical Association. Participants whose brain scanning included a susceptibility weighted imaging (SWI) sequence, as part of our previous study (Ren et al., 2020), were retrospectively recruited at the Movement Disorder Center of Neurology between January 2015 and July 2020. Clinical diagnoses of MSA-P and PD were made according to established criteria (Gilman et al., 2008; Postuma et al., 2015) by two clinicians with professional movement disorder experience of more than 10 years. In light of the consensus guidelines, our patients were clinical probable MSA-P and clinical definite PD, who were regularly followed-up every 6 months in our clinics.

Disease onset was defined by the occurrence of motor symptoms and the disease duration was defined as the period between the onset of motor symptoms and the date of the MRI. We selected patients who performed MRI with SWI sequence within 2 years of disease duration as early stage, according to a previous study (Oishi et al., 2009).

As a control group (CG), we selected the previously retrieved patients from the Picture and Communication System (PACS) using the same methods and exclusion criteria as reported in our previous study (Ren et al., 2020). Finally, 19 patients with MSA-P, 19 age- and sex- matched patients with PD with disease duration of no more than 2 years, and 10 CG participants were enrolled in this study.

### Scanning model and parameters

Axial scans were set parallel to the intercommissural line. Scanning parameters on the 3.0T MR scanner (Ingenia scanner, Philips Medical Systems, Netherlands) were as follows: slice thickness = 2 mm; TR = 20 ms; TE = 27 ms; flip angle: 15°; FOV = 220 mm; number of signal acquisitions: 1; and matrix size: 284 × 230.

### Region of interest and morphometric index extraction

**FIGURE 1:**
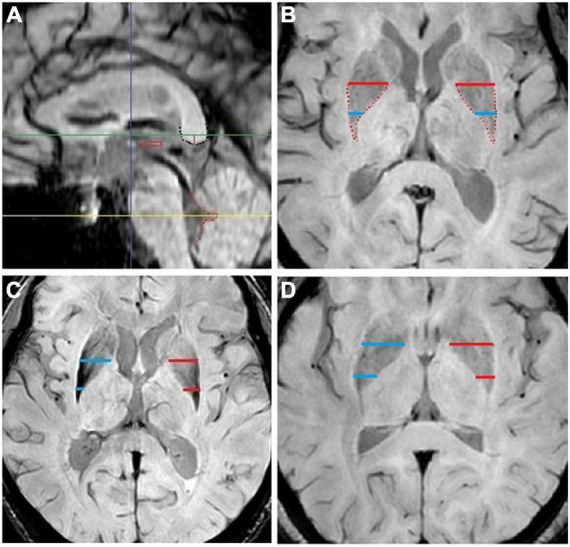
**(A)** Standard midsagittal plane. The thalamic syndesmosis (hollow red arrow), corpus callosum splenium (black dot) and 4th ventricle posterior edge (red dot). The parallel horizontal green and yellow line define the axis plane selection for lentiform nucleus and cerebrospinal fluid measurements: the green line is set at 3.5 mm (red vertical line) above the black dot, and the yellow line is set to cross the cusp of the red dot. The vertical blue line defines the anterior-posterior location of the lentiform nucleus short line and is set to cross the middle of thalamic syndesmosis. **(B)** Standard axis plane for lentiform nucleus measurement. The blue and red lines are used for the short and long lines of the lentiform nucleus measurement: the red line is the longest line near the middle area of the lentiform nucleus, and the blue line is defined by the vertical blue line in a. **(C)** F, 59, patient with probable multiple system atrophy-parkinsonian subtype with general weakness for 1 year, obviously in her lower limbs, walking drag, dizziness for 2 months, and turning over difficulty for 1 month. **(D)** F, 60, patient with Parkinson disease with an inflexible right upper limb for 2 years, right lower limb shake for 1 year, and the right upper limb shake for more than 2 months. The slice selection and measurement protocols were assigned according to our previous study. The smaller short line side (blue line in panel **C** and red line in panel **D**) was recorded as corrected short line (cSL), and the corrected ratio (cSLLr) was calculated using the long line with the same cSL side.

Two experienced radiologists defined standard axis plane, vertical line to define the anterior- posterior location of the LN in the PACS system according to our previous study as shown in Figures 1A,B (Ren et al., 2020). The longest horizontal line (LL), short line (SL), calculated SL/LL ratio (SLLr), area, mean signal intensity (SIm_LN), and standard deviation of the signal intensity (SIsd_LN) of the sketched boundary area of the LN were recorded in the magnitude image axis plane. The above indexes of both sides were recorded, and the uniformity of the two radiologists’ measurements were estimated by the mean value of both sides. The cerebrospinal fluid signal intensity of the fourth ventricle was also measured as SIm_CSF and SIsd_CSF. Then, the SIm_LN of each side was normalized to SIm_CSF with a signal intensity of 200 (nSIm), according to previous study (Meijer et al., 2012). For the above indexes, we calculated the mean value of the left and right value measured by each radiologist and then calculated the mean values of the two radiologists for the statistical analysis. According to our previous experience, patients with MSA-P are characterized by a narrowing morphology and inhomogeneous signal intensity of the posterior LN region. We revised the protocol and chose the smaller SL side (mean left side value of the two radiologists or right) as the corrected SL (cSL) and calculated the corrected ratio (cSLLr) by LL with the same cSL side, as shown in Figures 1C,D. We also chose the larger SIsd_LN side as the corrected cSIsd_LN for the following statistical analysis.

### Statistics

The Statistical Package for the Social Sciences (SPSS 22.0, Chicago, IL, United States) was used for the statistical analyses. Continuous variables are expressed as mean ± SD. One-way analysis of variance (ANOVA) with *post hoc* multiple comparisons conducted by least significant difference (LSD) was used for group and subgroup comparisons when variables conformed to a normal distribution by Shapiro–Wilk test; otherwise, a non-parametric test (Mann–Whitney U) was used. Statistical significance was defined as *P* < 0.05. Receiver operating characteristic (ROC) curves were plotted to assess the value of the significantly different index in differentiating MSA-P from PD and healthy controls, in which cutoff values were determined using the maximum Youden’s index (sensitivity + specificity-1). The intraclass correlation coefficient (ICC) was used to assess the uniformity of the two radiologists’ measurements.

## Results

### Patient demographics

The clinical and demographic characteristics of the participants are summarized in Table 1. No significant age and sex differences were observed between the three groups, and there were no differences in disease duration between patients with PD and MSA-P when the SWI scanned. Most of the follow up duration of the included patients (12/19) ranged from 4 to 8 years when we carried out this present study at June 2022, and the results were shown in Table 1.

**TABLE 1:** The demographic characteristic of multiple system atrophy-parkinsonian subtype, Parkinson’s disease and control group.

	MSA-P	PD	CG		*P*	
				
				MSA-P vs. PD	MSA-P vs. CG	PD vs. CG
Age (years, mean ± SD, range)	59.32 ± 7.97 (50–83)	60.32 ± 8.19 (48–83)	59.42 ± 7.92 (50–83)	0.703	0.968	0.733
Gender (Male/female)	10/9	10/9	10/9	1.000	1.000	1.000
Disease duration (months, mean ± SD, range)	17.05 ± 8.13 (3–24)	17.05 ± 8.75 (2–24)	NA	1.000	NA	NA
Follow up duration (months, mean ± SD, range)	55.79 ± 23.16 (20–91)	58.95 ± 23.61 (11–99)		0.680	NA	NA

### Uniformity of the double measurement results and inter-observer variability

The consistency of the left and right measured data differences between two radiologists was assessed using the ICC. The agreement level definitions based on ICC values were as follows: ICC < 0.3, slight agreement; ICC = 0.3–0.7, moderate agreement; ICC > 0.7, good agreement. The ICC values for patients with MSA-P were in good agreement and the best among the three groups, as shown in Table 2.

**TABLE 2:** The intraclass correlation coefficient of two radiologists’ measurement.

	SL	LL	SLLr	Area	SIm	SIsd	nSIm
PD	0.468	0.685	0.450	0.529	0.527	0.839	0.524
MSA-P	0.729	0.884	0.584	0.868	0.890	0.942	0.875
HC	0.702	0.607	0.510	0.531	0.521	0.593	0.472

### Comparison of the SL, LL, SLLr, Area, SIm, SIsd, and nSIm among the three groups

**FIGURE 2:**
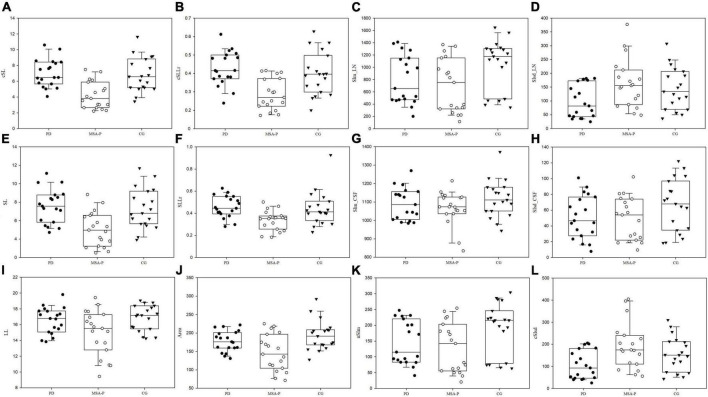
The box-scatter blot of the three groups with upper lower limit, upper lower quartile, and median line. The *x*-axis represents the three groups using different colors, and the *y*-axis represents the measured and calculated indexes. **(A)** corrected short line, cSL, **(B)** corrected short and long line ration, cSLLr, **(C)** mean signal intensity of lentiform nucleus, SIm_LN, **(D)** standard deviation of signal intensity of lentiform nucleus, SIsd_LN, **(E)** Short line, SL, **(F)** the ratio of short and long line, SLLr, **(G)** mean signal intensity of cerebrospinal fluid, SIm_CSF, **(H)** standard deviation of signal intensity of cerebrospinal fluid SIsd_CSF, **(I)** long line, LL, **(J)** Area, **(K)** normalized mean signal intensity, nSIm, and **(L)** corrected standard deviation of signal intensity, cSIsd.

There were no statistically significant differences in SIm_CSF, and SIsd_CSF, nSIm among the three groups. We found significant decreases in SL, cSL, SLLr, cSLLr and Area in the MSA-P group compared to the PD and CGs, a significant increase in SIsd_LN and cSIsd_LN in the MSA-P group compared to the PD group, and a significant decrease in the SIm_LN and nSIm in the MSA-P group compared to the CG. However, no significant difference was found between the MSA-P and PD groups of the SIm_LN and nSIm. The results are shown in Figure 2 and Table 3.

**TABLE 3:** Comparison of morphological and signal measurement among different group (mean ± standard deviation).

	MSA-P	PD	CG	*P*		
				
				MSA-P vs. PD	MSA-P vs. CG	PD vs. CG
cSL	4.22 ± 1.70	7.04 ± 1.76	6.81 ± 2.16	**0.000**	**0.000**	0.712
SL(mm)	5.07 ± 1.86	7.57 ± 1.84	7.29 ± 2.17	**0.000**	**0.001**	0.663
LL(mm)	14.97 ± 2.85	16.54 ± 1.66	16.86 ± 1.61	*0.099*	***0.037***	*0.422*
cSLLr	0.34 ± 0.09	0.46 ± 0.10	0.44 ± 0.15	**0.000**	**0.001**	0.398
SLLr	0.34 ± 0.09	0.46 ± 0.10	0.44 ± 0.15	**0.002**	**0.006**	0.725
Area(mm^2^)	146.78 ± 49.30	177.38 ± 28.31	193.42 ± 40.23	***0.049***	***0.010***	*0.314*
SIm_LN	698.13 ± 434.11	801.21 ± 397.56	1048.25 ± 410.81	0.447	**0.012**	0.072
SIsd_LN	164.13 ± 87.31	96.60 ± 59.52	145.09 ± 75.78	**0.008**	0.438	0.052
cSIsd_LN	191.23 ± 104.28	105.65 ± 64.64	155.97 ± 78.37	**0.003**	0.202	0.070
SIm_CSF	1066.31 ± 90.53	1089.97 ± 86.48	1118.57 ± 100.10	0.434	0.088	0.345
SIsd_CSF	49.02 ± 27.27	51.39 ± 27.54	66.23 ± 33.24	0.805	0.078	0.127
nSIm	130.43 ± 79.00	145.42 ± 69.02	191.39 ± 80.64	0.548	**0.017**	0.069

Significant P-values < 0.05 are highlighted in bold. The p-value in italics is the result of U test.

### Receiver operating characteristic curve analysis

**FIGURE 3:**
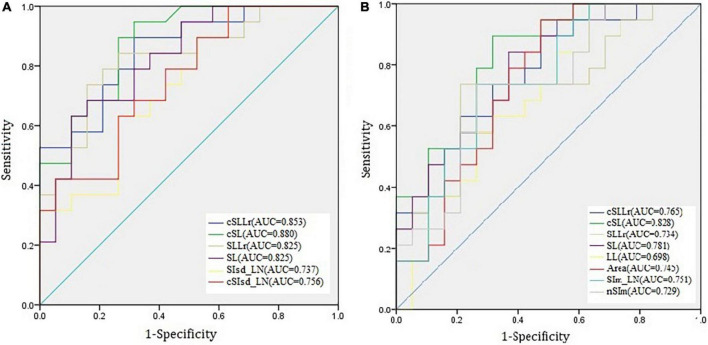
The receiver operating characteristic (ROC) curves of six indexes for multiple system atrophy-parkinsonian subtype vs. Parkinson’s disease **(A)** and eight indexes multiple system atrophy- parkinsonian subtype vs. control group **(B)**.

We performed ROC analysis based on the above six parameters, which were significantly different (*P* < 0.05) between MSA-P patients and PD, including cSL, SL, cSLLr, SLLr SIsd_LN, and cSIsd_LN; and eight parameters between MSA-P and CG, including cSL, SL, cSLLr, SLLr, Area, Sim_LN, and nSIm. The area under the curve (AUC) of the cSL was highest for differentiating MSA- P from PD and also for differentiating MSA-P from CG. Correspondingly the AUC was 0.880 for MSA-P vs. PD and 0.828 for MSA-P vs. CG, the sensitivity and specificity were 89.5 and 73.7% for MSA-P vs. PD, and 89.5 and 68.4% for MSA-P vs. CG, respectively. The results are shown in Figure 3.

## Discussion

We have attempted to distinguish MSA-P from PD using SWI in our previous study, combining the morphology and signal indexes of the LN, the sensitivity and specificity could reach 94.7 and 63.2% (Ren et al., 2020). In this study, we further verified the feasibility of this measurement protocol for early disease differentiation. The major findings were as follows: first, the measurable LN morphological changes could distinguish MSA-P from PD in early stage, with relative high sensitivity and moderate specificity. Second, the AUC of diametral indexes were higher than signal indexes for distinguishing MSA-P from PD. Third, the smaller SL side could improve the sensitivity and specificity than the mean value of the two sides.

Structural and functional MRI are widely used in clinic because of its high soft tissue resolution and various available tissue imaging parameters. In MSA-P, several characteristics on conventional MRI had been reported before, such as atrophy of the putamen; presence of a bilateral T2- hyperintense rim bordering the dorsolateral margins of the putamen; and T2-putaminal hypo- intensity (Saeed et al., 2017). VBM and volumetric studies typically reveal striatonigral involvement in MSA-P patients (Schulz et al., 1999; Tir et al., 2009). A recent meta-analysis has demonstrated that the putaminal volume significantly reduced in MSA versus PD patients (Sako et al., 2014). Notably, these signs have low sensitivity values and the appearance of these MRI markers can be influenced by image acquisition factors. However, combined analysis of biomarkers may better differentiate MSA-P from PD (Wadia et al., 2013; Feng et al., 2015). In MSA-P, an elevated mean diffusivity of the putamen was identified relative to PD, and healthy controls (Meijer et al., 2015b; Barbagallo et al., 2016). Remarkably, a combination of increased T2* relaxation rates and mean diffusivity in the putamen enabled discrimination of PD from MSA-P patients with high accuracy (Barbagallo et al., 2016). Likewise, Ito et al. (2007) found lower FA and increased apparent diffusion coefficient (ADC) values in MSA-P patients in putamen, cerebellum and pons, versus PD and controls. FA and ADC in the pons proved to be highly specific for differentiating MSA-P patients from PD (Ito et al., 2007). For metabolic alterations, lower NAA/Cr ratios in the putamen and pontine base best discriminated MSA-P cases from PD (Watanabe et al., 2004), but the result was opposite in another report (Tsuda et al., 2019). These above reports encourage us, but the manipulation methods used in the studies are complex and difficult to apply in the clinical context. In our study we want to investigate measurement of the morphological and intensity changes which could be used by clinicians to discriminate MSA-P from PD and controls.

Recent studies on the differential diagnosis between MSA-P and PD based on neuroimaging have shown encouraging findings. Regional apparent diffusion coefficients of middle cerebellar peduncles completely differentiated patients with MSA-P from those with PD, with a mean disease duration of 4.9 years (Nicoletti et al., 2006). In addition, the machine learning approach based on volumetry enabled accurate classification of subjects with early stage Parkinsonism with a mean disease duration of 5.0 years (Chougar et al., 2020). Proton magnetic resonance spectroscopy findings in the basal ganglia of patients with MSA-P with a mean disease duration of 3.4 years also differed from those of healthy controls (Stamelou et al., 2015). Although the patients with MSA- P in these studies were at Hoehn and Yahr stage ≤ 3 and thought to be at an early stage, there is a great difference between the progression rate and prognosis of PD and MSA-P. More than 50% of patients with MSA-P require walking aids within 3 years after the onset of motor symptoms, and 60% require a wheelchair after 5 years (Fanciulli and Wenning, 2015). This means that most patients with MSA-P are at H&Y stage ≥ 3 within 3 years, but patients with PD are still at a stage associated with good levodopa response and have no balance problems. Within 2 years of disease duration, it is very difficult to differentiate MSA-P and PD based on clinical manifestations. Other research papers used 2 years as an early stage to compare MSA-P and PD (Druschky et al., 2000; Oishi et al., 2009), and here, we compared the SWI of MSA-P and PD within 2 years to evaluate its usefulness for differential diagnosis. Using our measurement protocol, a relative high sensitivity of 89.5% and a moderate specificity of 73.7% could be achieved for distinguishing MSA-P from PD at early stages. Clinically available 3T conventional MRI contributes little to differentiating PD from atypical Parkinsonian disorders. The pathologic alterations of Parkinsonism show abnormal brain iron deposition; therefore, SWI, which is sensitive to iron concentration, has been applied to identify iron-related lesions for the diagnosis and differentiation of PD in recent decades (Wang et al., 2016). The LN anatomically includes the putamen and globus pallidus, and Wang et al. (2012) found that patients with MSA-P with a mean disease duration of 2.3 years had significantly higher iron deposition in the putamen compared to those with PD, but not in globus pallidus. The signal intensity of the bilateral posterior, dominant side of the posterior, mean values of the bilateral anterior, and posterior halves of the putamen on SWI differed significantly between MSA-P and PD, but there was much diversity in the course of the disease (Yoon et al., 2015). Furthermore, the signal in the putamen was significantly lower for patients with MSA-P with a disease duration of about 1.3 years than for those with PD with a similar disease duration and controls; thus, the ROI was selected in the posterior small region of the putamen (Meijer et al., 2015a). In the present study, we enrolled subjects within 2 years of the disease course and found that the mean signal intensity changes of the whole LN posterior portion did not have sufficient significance to distinguish MSA-P from PD at an early stage. Although the SIsd_LN and cSIsd_LN were significantly higher in the MSA-P group than in the PD group, this index could not distinguish MSA-P from controls. Thus, the signal intensity changes of the whole posterior LN position were insufficient markers for the early differential diagnosis of MSA-P, although the posterior-dorsal part of the putamen signal intensity may be helpful.

There are limited studies on morphological changes in MSA-P, such as on different brain structure volumes, machine learning algorithms (Fanciulli and Wenning, 2015), subjective putamen atrophy (Meijer et al., 2015b), striatal volumes-of-interest (VOIs) 123 I-FP-CIT uptake, and support vector machine analysis (Nicastro et al., 2019). The related prior studies considered the morphological change in MSA-P to be a useful marker for the differential diagnosis of PD. MSA-P typically presents with relatively more symmetrical Parkinsonism than PD (Gilman et al., 2008). It is interesting to note that there was a relatively asymmetrical measurement of the morphological changes, especially the index of SL. Using the smaller side of the SL (cSL), rather than the mean value of the two sides, can improve the sensitivity and specificity, the AUC of cSLLr increased from 0.825 to 0.853 compared to SLLr. As reported in the previous pathological report, slicing the brain reveals atrophy and dark brownish discoloration of the posterolateral putamen due to deposition of lipofuscin, neuromelanin, and increased iron content in this area (Dexter et al., 1992), and one of the histological core features of MSA including selective neuronal loss and axonal degeneration involving multiple regions of the nervous system with brunt on the striatonigral and olivopontocerebellar systems (Jellinger, 2018). Our finding may further suggest that iron accumulation area be narrowed and may indicate cell loss and atrophy of the LN.

Several limitations of our primary and exploratory study should be noted: first, a pathological diagnosis has not been obtained in the present study. Second, the sample size was relatively small because of the low incidence of MSA-P, and the fact that this was a single-center study. Third, the retrospective study did not relate morphological and signal changes to the patients’ clinical scales, such as the Unified Multiple System Atrophy Rating Scale (UMSARS) and the motor and activities of daily living (ADL) scale. Fourth, The ICCs of PD and CG were often moderate consistent with the potential reason of the lower signal intensity of LN in MSA-P which make the boundary in MSA-P was easier to identify. Fifth, it’s hard to differentiate the globus pallidus and the putamen boundary in the magnitude image, so we chose the boundary of LN for the measurements, and this would dilute the results of putaminal iron change. Further randomized multi-centric studies with larger sample sizes are required in the future.

Despite these limitations, we further verified the feasibility of this objective measurement in the early disease duration based on our previous work. We found that the patients with MSA-P were characterized by a narrowing of the posterior region of the LN, compared to those of the PD and CGs, which might help physicians differentiate MSA-P from PD at early stages.

## Data availability statement

The original contributions presented in this study are included in the article/supplementary material, further inquiries can be directed to the corresponding author/s.

## Ethics statement

The studies involving human participants were reviewed and approved by the Ethics Committee of Qilu Hospital. The ethics committee waived the requirement of written informed consent for participation.

## Author contributions

QR made the measurements and finished the statistical analysis, and draft and submitted the manuscript. CZ made the study concept, MSA-P and PD diagnose and revised the manuscript. YW revised the manuscript. JZ made the MSA-P and PD diagnose, made the measurements, and collected the controls data. XM made the interpretation of the results. XX revised the manuscript. All authors contributed to the article and approved the submitted version.

## Abbreviations

SWI: susceptibility weighted imaging
LN: lentiform nucleus
MSA: multiple system atrophy
MSA-P: multiple system atrophy-parkinsonian subtype
MSA-C: multiple system atrophy cerebellar subtype
PD: Parkinson’s disease
CG: control group
PACS: Picture and Communication System
LL: longest horizontal line
SL: short line
SLLr: the ratio of SL/LL
SIm_LN: mean signal intensity of lentiform nucleus
SIsd_LN: standard deviation of signal intensity of lentiform nucleus
nSIm: normalized SIm_LN
cSL: corrected SL
cSLLr: corrected SLLr
ANOVA: One-way analysis of variance
LSD: Least Significant Difference
ROC: Receiver operating characteristic
ICC: Intraclass correlation coefficient
AUC: area under the curve
VOIs: volumes-of-interest
UMSARS: Unified Multiple System Atrophy Rating Scale
ADL: activities of daily living.
